# Real-time TIRF observation of vinculin recruitment to stretched α-catenin by AFM

**DOI:** 10.1038/s41598-018-20115-8

**Published:** 2018-01-25

**Authors:** Koichiro Maki, Sung-Woong Han, Yoshinori Hirano, Shigenobu Yonemura, Toshio Hakoshima, Taiji Adachi

**Affiliations:** 1 Laboratory of Biomechanics, Department of Biosystems Science, Institute for Frontier Life and Medical Sciences, Kyoto University, 53 Shogoin-Kawahara-cho, Sakyo, Kyoto 606-8507 Japan; 2 Department of Micro Engineering, Graduate School of Engineering, Kyoto University, Yoshida Honmachi, Sakyo, Kyoto 606-8501 Japan; 3 National Institute for Nanomaterials Technology, Pohang University of Science and Technology, 77 Cheongam-ro, Nam-Gu, Pohang, Gyeongbuk 790-784 Korea; 4 Structural Biology Laboratory, Graduate School of Biological Sciences, Nara Institute of Science and Technology, 8916-5 Takayama, Ikoma, Nara 630-0192 Japan; 5 Department of Cell Biology, Graduate School of Medical Science, Tokushima University, 3-18-15 Kuramoto-cho, Tokushima, Tokushima, 770-8503 Japan

## Abstract

Adherens junctions (AJs) adaptively change their intensities in response to intercellular tension; therefore, they integrate tension generated by individual cells to drive multicellular dynamics, such as morphogenetic change in embryos. Under intercellular tension, α-catenin, which is a component protein of AJs, acts as a mechano-chemical transducer to recruit vinculin to promote actin remodeling. Although *in vivo* and *in vitro* studies have suggested that α-catenin-mediated mechanotransduction is a dynamic molecular process, which involves a conformational change of α-catenin under tension to expose a cryptic vinculin binding site, there are no suitable experimental methods to directly explore the process. Therefore, in this study, we developed a novel system by combining atomic force microscopy (AFM) and total internal reflection fluorescence (TIRF). In this system, α-catenin molecules (residues 276–634; the mechano-sensitive M_1_-M_3_ domain), modified on coverslips, were stretched by AFM and their recruitment of Alexa-labeled full-length vinculin molecules, dissolved in solution, were observed simultaneously, in real time, using TIRF. We applied a physiologically possible range of tensions and extensions to α-catenin and directly observed its vinculin recruitment. Our new system could be used in the fields of mechanobiology and biophysics to explore functions of proteins under tension by coupling biomechanical and biochemical information.

## Introduction

Adhesive interaction between neighboring cells contributes to the mechanical maintenance of morphogenetic changes in embryos as well as homeostasis in mature tissues^1–3^. The dysfunction of proteins involved in intercellular adhesion thus causes a variety of pathologies^4–6^. Cadherin-based adherens junctions (AJs) connect the actin cytoskeleton between cells and dynamically change the strength of adhesion in response to intercellular tension^7–12^. During AJ maturation, α-catenin and vinculin cooperate to remodel the actin cytoskeleton^13–15^. α-Catenin participates in the cadherin-catenin complex (CCC), whereas cadherin, β-catenin, and α-catenin serially associate together^16–18^. The CCC interacts with actin filaments via α-catenin in the cytoplasmic region of AJs to transmit intercellular tension generated by the actomyosin cytoskeleton^19,20^. Under intercellular tension, α-catenin recruits vinculin^13^, which is another key protein in the architecture of AJs. The C-terminus of vinculin interacts with monomeric and filamentous actin to mediate the link between α-catenin and another actin filament^21^. α-Catenin and vinculin thereby induce local remodeling of the actin cytoskeleton at AJs to increase the strength of adhesion^22^, which stably transmits intercellular tension.

α-Catenin under tension-free conditions is known to form an autoinhibited conformation against vinculin^23,24^; the vinculin binding site (VBS, residues 325–360) is embedded in the M_1_ domain (residues 273–393) of α-catenin, which is further stabilized by the M_2_-M_3_ domain (residues 394–634)^25^. Thus, under tension, it has been suggested that the autoinhibitory interaction between the M_1_ domain and the M_2_-M_3_ domain is disrupted, and the destabilized M_1_ domain exposes the VBS to recruit vinculin^13^. Our previous report^26^ has revealed that, using atomic force microscopy (AFM)-based single-molecule force spectroscopy, the mechanical disruption of the autoinhibitory M_1_/M_2_-M_3_ interaction induces a drastic change in the conformation of α-catenin, together with increased mechanical stability. In addition, another single-molecule study showed that the affinity of α-catenin for the head domain of vinculin increases under tension^27^. However, although the α-catenin-mediated mechanotransduction is a dynamic molecular process, there is no experimental technique at the molecular level that allows us to observe vinculin recruitment to α-catenin under tension in real time.

Therefore, in the present study, we developed a system combining AFM and total internal reflection fluorescence (TIRF) to directly observe the dynamic recruitment of vinculin to α-catenin. AFM-based techniques, such as DNA cutting/pasting^28^ and structural imaging^29^, have been combined with TIRF-based molecular observation to investigate biomechanical phenomena at the molecular level. Using the new system, we extended α-catenin molecules (the mechano-sensitive M_1_-M_3_ domain; residues 276–634) using AFM and simultaneously observed their recruitment of full-length vinculin molecules in real time using TIRF. We used full-length vinculin in the experiment to minimize tension-free binding of vinculin to α-catenin, as it forms an autoinhibited conformation against α-catenin^30–32^. A previous study based on isothermal titration calorimetry (ITC) reported no detectable interaction between the α-catenin M_1_-M_2_ domain, which is an open form fragment without the autoinhibitory M_3_ domain, and full-length vinculin^33^. In our study, we hypothesized that the mechanical force drastically destabilizes the M_1_ domain of α-catenin to allow its interaction with full-length vinculin.

## Results

### Mechanically-induced conformational change of α-catenin

**Figure 1:**
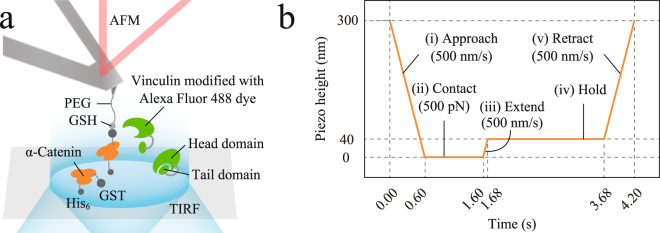
Schematic of atomic force microscopy (AFM) combined with total internal reflection fluorescence (TIRF). (**a**) α-Catenin molecules (M_1_-M_3_ domain, residues 276–634, and M_2_-M_3_ domain, residues 385–634), with a GST-tag at the N-terminus and a His_6_-tag at the C-terminus, were chemically modified on coverslips at the C-terminus and were mechanically loaded using an AFM tip; simultaneously, full-length vinculin molecules (residues 1–1066), dissolved in solution, were observed by TIRF. Vinculin molecules modified with Alexa Fluor 488 dye were illuminated when they came into in an evanescent field in TIRF (cyan region, approximately 150 nm from the surface of coverslips). (**b**) Movement of the base of the AFM cantilever was regulated by a programmed piezo-height as follows: (i) Decrease the piezo-height with the velocity of 500 nm/s to approach the AFM tip to the α-catenin-modified coverslip and stop when the reaction force reaches 500 pN (the piezo-height is set to zero at this point); (ii) keep the piezo-height to wait for an interaction between the AFM tip and α-catenin; (iii) increase the piezo-height by 40 nm with a velocity of 500 nm/s to extend α-catenin; (iv) keep the piezo-height constant at 40 nm to hold α-catenin under tension and wait for vinculin recruitment; and (v) increase the piezo-height by 260 nm with the velocity of 500 nm/s to completely retract from the coverslip and examine the resistance force of α-catenin with or without vinculin recruitment.

In our novel system, we extended α-catenin molecules (residues 276–634; the mechano-sensitive M_1_-M_3_ domain), modified on coverslips, using AFM and simultaneously observed their recruitment of Alexa-labeled full-length vinculin molecules, dissolved in solution, using TIRF (Fig. 1a). α-Catenin molecules attached to coverslips were tethered by AFM in programmed piezo-height control phases (Fig. 1b).

**Figure 2:**
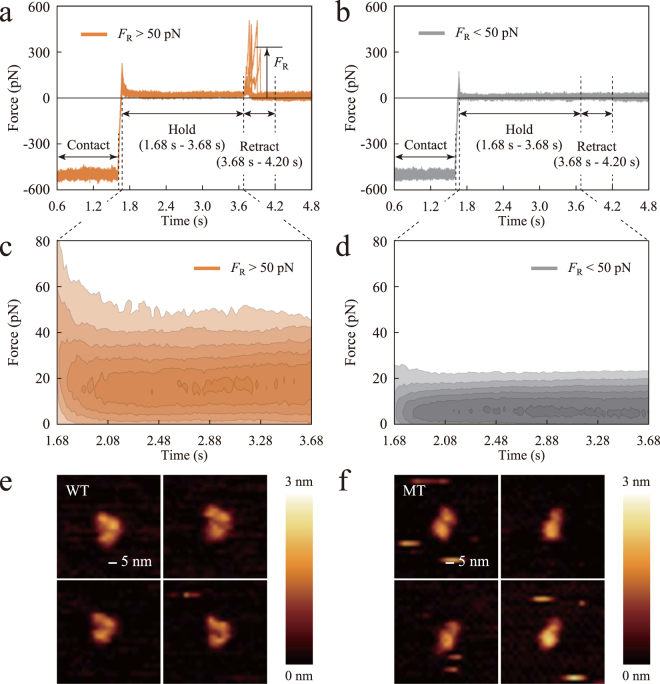
AFM-based extension experiment and structural imaging for α-catenin. (**a**,**b**) Force versus time curves from the contact phase. Tensile force is shown as positive and five individual force curves are overlaid together. Force curves were classified as (**a**) those with successful α-catenin extension; and (**b**) those without α-catenin extension, based on the value of rupture force, *F*_R_, as indicated by an arrow. (**c**,**d**) Contour maps of number densities of force-time data points in the hold time in each group. Color contours are set from 0.2 times (light) to 0.85 times (dark) of the maximum value of the number density. (**e**,**f**) Results of AFM structural imaging. The images show (**e**) the autoinhibited conformation of the wild-type α-catenin fragment and (**f**) the opened conformation of the mutated fragment.

As a result of the AFM-based α-catenin extension experiment, we obtained force curves (force versus time curves) as shown in Fig. 2a,b. In both figures, the tensile force is shown as positive and five force curves are overlaid together. The time course is set from 0.6 s, i.e., from the contact phase in piezo-height control. The force curves were classified into two groups: with successful α-catenin extension (Fig. 2a) and without (Fig. 2b), based on the rupture force, *F*_R_, observed in the retract phase. The threshold for *F*_R_ (50 pN) for the classification was determined by comparison of the results of the control extension experiment by AFM without α-catenin modification onto coverslips (see Supplementary Fig. 1). In our experiment, approximately 52.6% of the force curves (2264 curves/total 4305 curves) showed successful α-catenin extension and the remainder (approximately 47.4%) did not. Thereby, force curves with successful α-catenin extension showed approximately 15 pN of force during the hold phase (Fig. 2c), which was much higher than that without α-catenin extension (Fig. 2d). The contour maps in Fig. 2c,d represent the number density of force-time data points in all the curves in each group. The approximately 15 pN force found in the curves with α-catenin extension (Fig. 2c) would be sufficient to open the autoinhibition of α-catenin^27^ and were within the physiological range^34^. We also suggest an approximately 5 pN force observed in the curves without α-catenin extension (Fig. 2d) was caused by non-specific tethering in some curves. Thus, using AFM, we mechanically opened the conformation of α-catenin to allow its recruitment of vinculin during the hold time.

To explore the conformational change of α-catenin under tension, we performed an AFM-based structural imaging experiment. In this experiment, we tested wild-type and mutant (M319G and R326E) M_1_-M_3_ domains of α-catenin. The mutated fragment, possibly with an attenuated intra-domain interaction between the M_1_ and M_2_-M_3_ domains, was reported to recruit full-length vinculin in a tension-insensitive manner^26^. We validated that the labeling of full-length vinculin by Alexa-Fluor 488 dye did not affect its affinity for α-catenin. As a result of the AFM structural imaging, we obtained three-dimensional features of the α-catenin fragments, as shown in Fig. 2e,f. Wild-type α-catenin (Fig. 2e) exhibited three regions corresponding to three helix bundles, as proposed in a previous structural biology study^23,24^. By contrast, the mutated α-catenin (Fig. 2f) had a different configuration, with one region having a similar size to each domain in the wild-type as well as possessing another larger region. As the M_2_ and M_3_ domains of α-catenin are known to get close to each other by rotation in the absence of the M_1_ domain^35^, we concluded that the larger region corresponds to the rotated M_2_ and M_3_ domains getting close to each other, and that the smaller region corresponds to the M_1_ domain. This result implied that residues 276–634 of α-catenin, with an attenuated M_1_/M_2_-M_3_ interaction under tension, change their conformation to expose the VBS-containing M_1_ domain, which then enables interaction with full-length vinculin.

### Real-time TIRF observation of vinculin recruitment to stretched α-catenin by AFM

**Figure 3:**
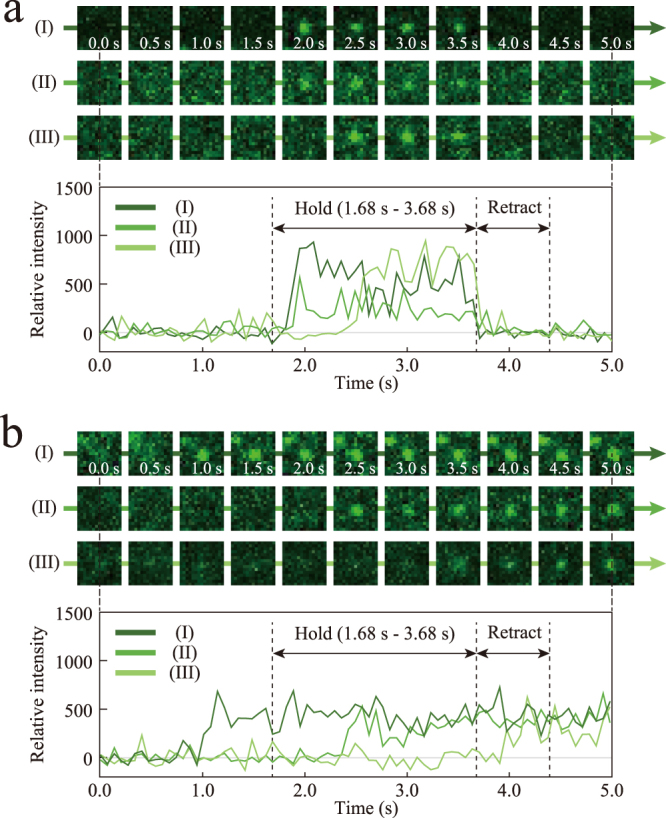
Real-time TIRF observation of vinculin recruitment to stretched α-catenin by AFM. (**a**) Square pulses with increase in intensity after 1.68 s, representing vinculin recruitment to α-catenin under tension and a decrease at 3.68 s, representing vinculin dissociation caused by further unfolding of α-catenin. The time courses of relative intensity were shown in the bottom part of the panel. (**b**) Step increases in intensity, independent of the timing of AFM experiment, which was caused by nonspecific vinculin interaction with the coverslip.

The dynamics of full-length vinculin molecules were observed by TIRF simultaneously with measurement of the extension of α-catenin by AFM. In movies obtained in conjunction with successful α-catenin extension, we observed a square pulse in the intensity (Fig. 3a) with a possibility of approximately 0.71% (16 events/2264 events), in which the intensity increased during the hold phase and then decreased at the beginning of the retract phase (see Supplementary Material, Video S1). This result indicated that full-length vinculin interacted with α-catenin under approximately 15 pN tension and then dissociated upon further extension of α-catenin. Similar square pulses were observed with a possibility of approximately 0.10% (2 events/2041 events) in the movies obtained without successful α-catenin extension; however, the timing of the decrease in the intensity was not synchronized with the force measurement. These unsynchronized square pulses could be caused by transient association/dissociation of vinculin with α-catenin in the autoinhibited conformation under no tension. In movies either with or without successful α-catenin extension, we observed a different type of signal (Fig. 3b), that is, a step increase in the intensity (see Supplementary Material, Video S2) with a possibility of approximately 0.75% and approximately 0.59% (17 events/2264 events and 12 events/2041 events), in which the intensity increased independently of the hold phase and was sustained until each AFM measurement was completed. We further determined that the step increases resulted from nonspecific vinculin interaction with the surface of the coverslip, not to α-catenin molecules, as we observed the step increases on coverslips without α-catenin modification (any other chemical compounds were modified in the same way). We also confirmed that the intensity in step increases was sustained for more than 20 s in longer observations, supporting that the drop in the intensity in square pulses was not caused by photo-bleaching but caused by the dissociation of vinculin from α-catenin. Furthermore, the square pulses were not observed when we used the M_2_-M_3_ domain (residues 385–634), without the M_1_ domain. This result indicated that vinculin interacted with the M_1_ domain of α-catenin but did not interact with the M_2_-M_3_ domain or any linker components. Thus, using this novel AFM-TIRF combined system, we could directly observe the recruitment of full-length vinculin to α-catenin under tension at the molecular level.

### Conformational stabilization of α-catenin by vinculin recruitment

**Figure 4:**
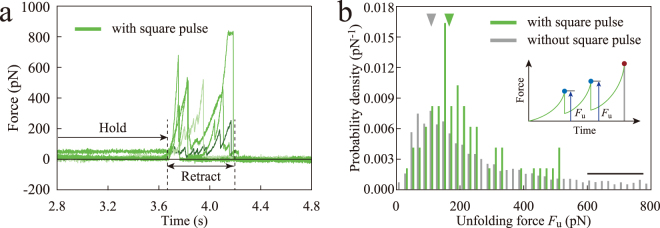
Stabilization of α-catenin by vinculin recruitment. (**a**) Force curves in conjunction with square pulses (i.e., vinculin recruitment) observed by TIRF. (**b**) Histogram of unfolding force (*F*_u_) for force curves in conjunction with and without square pulses observed by TIRF. *F*_u_ was measured at each peak except the final peak (rupture peak) in the hold time.

To understand how vinculin recruitment changes the force response of α-catenin, we analyzed the force curves in conjunction with the square pulses observed by TIRF, i.e., with vinculin recruitment (Fig. 4a). In this analysis, the force curves with successful α-catenin extension (2264 curves) were further classified into two groups: Curves with square pulses (16 curves) and those without (2248 curves). We then measured the unfolding force, *F*_u_, in the retract phase (Fig. 4b), which indicates the force response of α-catenin with and without vinculin recruitment, at every peak in the force curves, except for the last peak, which was caused by the detachment of the AFM tip from α-catenin. The histogram of the *F*_u_ for the force curves with square pulses (green bars, Fig. 4b) showed a higher mode value of approximately 180 pN, compared with approximately 100 pN for the force curves without square pulses (gray bars, Fig. 4b). This result indicated that α-catenin in an open conformation under tension is further mechanically stabilized by vinculin recruitment, which was consistent with the previous observations^26,27^. The high *F*_u_ of more than 600 pN in the force curves without square pulses (black bar, Fig. 4b), which we did not observe in our previous study^26^, could be caused by parallel fishing of several α-catenin molecules; we adopted a 50-fold higher concentration of α-catenin in this study compared to our previous single molecule study to increase the possibility of obtaining an interaction between the AFM tip and α-catenin molecules. Thus, by analyzing the force curves in the retract phase, we confirmed that the square pulses observed in TIRF corresponded with vinculin association/dissociation with α-catenin under tension.

## Discussion

Using the new AFM-TIRF system, we directly observed the recruitment of full-length vinculin to the M_1_-M_3_ domains of α-catenin under tension. In the context of mechanobiology, certain proteins have been identified as mechano-chemical transducers or mechano-sensors, which expose a cryptic binding site under mechanical force to recruit other proteins^13,36–39^. However, the identification and characterization of mechano-sensor proteins is still at the development stage because of the lack of suitable experimental approaches to directly detect the interaction between proteins under force. Our new system can be used in the fields of mechanobiology and biophysics to unravel the functions of proteins under tension by coupling biomechanical and biochemical information.

From our results, vinculin dissociation events were observed even in cases where the α-catenin was not fully unfolded in the retract phase, i.e. the α-catenin extensions at the rupture events were shorter than its fully extended length of approximately 143 nm. This result suggests that vinculin dissociated from α-catenin at the beginning of the retract phase (from 3.68 s to 4.20 s). This result is consistent with our previous AFM measurement^26^, in which full-length vinculin-bound α-catenin showed large *F*_u_ with small extensions (approximately 29.4 nm), suggesting that vinculin dissociated from α-catenin under this small amount of extension. Although the isolated head domain of vinculin is reported to dissociate only when the α-catenin M_1_-M_3_ domain is almost fully extended^27^, our result suggested that full-length vinculin dissociates more readily from α-catenin VBS than does the vinculin head domain. We suggested that this difference was caused by the autoinhibitory interaction in the full-length vinculin; the tail domain of vinculin competed with α-catenin VBS to interact with the head domain of vinculin under our conditions, which made the vinculin head domain easier to dissociate from α-catenin VBS under high tension. Our results also suggested that full-length vinculin dissociates easily from α-catenin when the vinculin affinity for α-catenin decreases under attenuated tension, with recovery of the autoinhibited conformation^40^.

The actual extension of α-catenin should also be discussed. We analyzed the tip-coverslip distance by moving the average and deviation in the tip-coverslip distance, which differed by approximately 15 nm in the force curves with and without successful α-catenin extension (see Supplementary Fig. 2). This value was reasonable because the AFM cantilever was deflected by approximately 25 nm to the inverse (pushing) direction at the wait phase. When the piezo-height increased to 40 nm at the extend phase, the AFM cantilever deflected to the pushing direction first went back to the original position, with an increase in piezo-height of approximately 25 nm, and then deflected to the extending direction with an increase in piezo-height of approximately 15 nm. Thereby, the curves without α-catenin extension (gray line, Supplementary Fig. 2), most of which did not show even nonspecific tethering, showed approximately 15 nm of the tip-coverslip distance. The reason why the group with α-catenin extension (orange line, Supplementary Fig. 2) also showed approximately 15 nm of the tip-coverslip distance would be that the cantilever stiffness was much higher than that of α-catenin or other tethered components, such as polyethylene glycol (PEG). Although it is difficult to estimate the absolute value of extension in α-catenin, since the other components, such as PEG, were also extended like α-catenin in series, and the length of PEG varied, we emphasize that the force range of approximately 15 pN was sufficient to open the α-catenin conformation, as validated by the previous reports^26,27^.

The number of molecules tested in this study needs to be discussed. In these experiments, we needed to increase the concentration of α-catenin on coverslips so that the possibility of an α-catenin-extending event could be increased to approximately 50%. As a result, it is possible that we occasionally parallel-loaded a couple of α-catenin molecules at the same time, because we observed a long tail in the unfolding force histogram in Fig. 4b. This parallel loading could also have been caused by the presence of a glutathione-S-transferase (GST)-tag at the N-terminus of α-catenin, as the GST-tag itself can form a dimer. Therefore, our results cannot be exclusively interpreted as single-molecule events and therefore this might cause a variation in the configuration of the force curves. Nevertheless, during AFM structural imaging, the area corresponding to α-catenin molecules showed a symmetric distribution (see Supplementary Fig. 3), suggesting that most α-catenin molecules existed as monomers in the same buffer used in our AFM-TIRF experiment. We also verified that a certain amount of full-length vinculin molecules can exist as monomers by cause of dithiothreitol (DTT) dissolved in AFM-TIRF experiment (see Supplementary Fig. 4), but the remainder of the molecules formed dimers, trimers, or even higher order oligomers. This analysis suggests that vinculin exists as a self-associated complex, as well as a monomer, in cells. In future work, it would be interesting to observe the effect of vinculin self-association on the interaction between α-catenin and vinculin under tension.

## Methods

### Protein purification

α-Catenin and vinculin were expressed and purified as reported in our previous study^26^. DNA fragments of mouse αE-catenin M_1_-M_3_ (residues 276–634) and M_2_-M_3_ (residues 385–634) were amplified by PCR and cloned into the pGEX6P-3 vector (GE Healthcare). A DNA fragment of mouse full-length vinculin (residues 1–1066) was cloned into the pET-6b (+) vector (Novagen). These plasmids were verified by DNA sequencing and transformed into *Escherichia coli* strain BL21Star (DE3) cells (Invitrogen). α-Catenin and vinculin molecules were expressed at 20 °C in Luria-Bertani medium supplemented with 0.1 mM isopropyl-β-d-thiogalactopyranoside. BL21Star cells expressing α-catenin and vinculin were suspended in 20 mM Tris-HCl buffer (pH 8.0) containing 150 mM NaCl and disrupted by sonication. The supernatant after ultracentrifugation was applied to a Glutathione Sepharose 4B column (GE Healthcare). Proteins eluted from the column were further purified by anion exchange (HiTrap Q HP, GE Healthcare) and gel filtration (Superdex 200 pg, GE Healthcare) chromatography. N-terminal His_6_ tags on vinculin molecules were then cleaved using human rhinovirus 3C protease. For AFM structural imaging, GST-tags at N-termini of the wild-type and mutated fragments were cleaved by PreScission Protease (GE Healthcare).

### Chemical modification for coverslips and AFM tips

Coverslips (Matsunami Glass Ind. Ltd.) and silicon nitride AFM tips (OMCL-TR400PSA; spring constant, 0.02 N/m; curvature radius of tip, 15 nm; Olympus Co.) were chemically modified for use in AFM (Nanowizard3 Ultra; JPK Instruments AG) as described in a previous study^26^.

To make a chamber to contain the working buffer with vinculin, we placed flat silicon rings on coverslips. The coverslips were cleaned in a plasma cleaner, sonicated in 1 M KOH for 15 min, and then sonicated in ethanol for 15 min. After cleaning, the coverslips were thoroughly washed in MilliQ-treated water (EMD Millipore, Hayward, CA, USA). For modification with α-catenin molecules, the coverslips were treated with 2% MPTMS/ethanol for 15 min and then treated with 2 mM maleimide-C_3_-NTA (Mal-C_3_-NTA; DOJINDO Laboratories, Kumamoto, Japan) in 20 mM Tris-HCl buffer for 30 min. The NTA-modified coverslips were then treated with 10 mM NiCl_2_ (Wako Pure Chemical Industries, Osaka, Japan)/MilliQ water for 30 min and further washed with MilliQ water and 20 mM Tris-HCl buffer. Using NTA-Ni^2+^-His_6_ affinity binding, 500 μM α-catenin fragments in 20 mM Tris-HCl buffer were immobilized on the coverslips. The coverslips were finally washed with 20 mM Tris-HCl buffer.

The AFM tips were first cleaned in a plasma cleaner and then treated with 2% APTES/MilliQ for 15 min. The tips were then treated with 6 mM Mal-PEG-NHS ester/20 mM Tris-HCl buffer for 30 min and with 10 mM glutathione/20 mM Tris-HCl buffer for 1 h. The remaining maleimide groups were quenched with 50 mM 2-mercaptoethanol/MilliQ water and washed with 20 mM Tris-HCl buffer.

### Fluorescent labeling of vinculin

Full-length vinculin molecules were labeled using the Alexa Fluor 488 Protein Labeling Kit (Thermo Fisher Scientific, Waltham, MA, USA), in which the primary amines in vinculin formed covalent bonds with the Alexa 488 dye. Full-length vinculin molecules and the Alexa 488 dye were conjugated at a 1:1 ratio at room temperature. Alexa-488-labeled vinculin molecules were purified using size exclusion chromatography. For each measurement using TIRF-combined AFM, Alexa 488-labeled vinculin molecules were dissolved at a concentration of 10 nM in working buffer (20 mM Tris-HCl pH 8.0, 1 mM DTT, and 2 mM Trolox). DTT was added to the working buffer to prevent aggregation of vinculin molecules caused by disulfide bonds, and Trolox was used to sustain the fluorescence of the Alexa 488 dye by maintaining reducing conditions in the working buffer.

### Settings for TIRF combined with AFM

For the AFM-TIRF combined system, we first calibrated the actual force measured by AFM. We adjusted the laser position to the top of cantilever in the working buffer using a non-oil 20x magnification lens and approached the bare coverslip to evaluate the relationship between the change in voltage from the photodiode and the deflection of the cantilever. The deflection was then calibrated to force based on the spring constant of the AFM cantilever. After calibration, we moved the base of the cantilever upward by 1000 nm and then removed the AFM head unit. The objective lens was then switched to a 100x objective lens for TIRF (UAPON 100XOTIRF; NA, 1.49; Olympus Co.) and the α-catenin-modified coverslip was set on the stage and the space inside the silicon ring was filled with the working buffer containing vinculin. We set up the TIRF condition by controlling the optional axis of the 488-nm laser (OBIS 488 LS; Coherent, Inc., Santa Clara, CA, USA), so that the penetration depth of the evanescent field was 150 nm, which covered the length of a fully extended αE-catenin M_1_-M_3_ fragment (approximately 143 nm). After focusing on the surface of the coverslip, the AFM head unit was brought back to the stage and the AFM tip was directed towards the coverslip surface. The power of the laser was first set at maximum for 30 min to diminish the fluorescence of any vinculin that was nonspecifically attached to the surface, and then the power was decreased to 2.97 W/cm^2^ using an ND filter to allow for AFM-TIRF measurement under conditions that avoided photo-bleaching. During the AFM force measurement, the fluorescence of vinculin was observed using an EM-CCD camera (iXon Ultra 888; Andor Technology Ltd., Belfast, UK); the exposure time was 50 ms and the final frame rate was approximately 14.76 frames/s. To synchronize the force measurement with the vinculin observation, we used the pulse signal output from the AFM controller that then externally controlled the EM-CCD camera. Finally, we analyzed the intensity of fluorescence in the square region (15 × 15 pixels) around the position of the tip.

### AFM structural imaging

For the AFM structural imaging, a fresh mica surface was obtained by peeling off the surface layers using tape. Wild-type and mutant α-catenin fragments (1 nM of each) in the working buffer (50 μL) were dropped onto the mica surface and allowed to absorb for 1 h. The solution was then washed using working buffer, and finally fresh working buffer (50 μL) was dropped onto the surface. Silicon AFM tips specialized for structural imaging (OMCL-AC160TS; spring constant, 26 N/m; resonant frequency, 300 kHz; curvature radius of tip, 7 nm; Olympus Co.) were used to obtain the images.

## Electronic supplementary material

Supplementary information

Supplementary Video S1

Supplementary Video S2
