# Supplementary material for: Real-time TIRF observation of vinculin recruitment to stretched α-catenin by AFM
Source: Sci Rep. 2018 Jan 25;8:1575. doi: 10.1038/s41598-018-20115-8 (PMC5785519; doi:10.1038/s41598-018-20115-8)
Supplement: Supplementary file 1 — Supplementary information [file 41598_2018_20115_MOESM1_ESM.pdf]

Supplementary Information

Real-time TIRF observation of vinculin recruitment to stretched α-catenin by AFM

Koichiro Maki[1,2], Sung-Woong Han[3], Yoshinori Hirano[4],

Shigenobu Yonemura[5], Toshio Hakoshima[4], Taiji Adachi[1,2]

[1]Laboratory of Biomechanics, Department of Biosystems Science, Institute for Frontier Life and Medical Sciences, Kyoto University, 53 Shogoin-Kawahara-cho, Sakyo, Kyoto 606-8507, Japan

[2]Department of Micro Engineering, Graduate School of Engineering, Kyoto University, Yoshida Honmachi, Sakyo, Kyoto 606-8501, Japan

[3]National Institute for Nanomaterials Technology, Pohang University of Science and Technology, 77 Cheongam-ro, Nam-Gu, Pohang, Gyeongbuk 790-784, Korea

[4]Structural Biology Laboratory, Graduate School of Biological Sciences, Nara Institute of Science and Technology, 8916-5 Takayama, Ikoma, Nara 630-0192, Japan

[5]Department of Cell Biology, Graduate School of Medical Science, Tokushima University, 3-18-15 Kuramoto-cho, Tokushima, Tokushima 770-8503, Japan

*Corresponding author:

T. Adachi, Laboratory of Biomechanics, Department of Biosystems Science, Institute for Frontier Life and Medical Sciences, Kyoto University, 53 Shogoin-Kawahara-cho, Sakyo, Kyoto 606-8507, Japan

Tel.: +81-75-751-4853; Fax: +81-75-751-4853; E-mail: adachi@frontier.kyoto-u.ac.jp

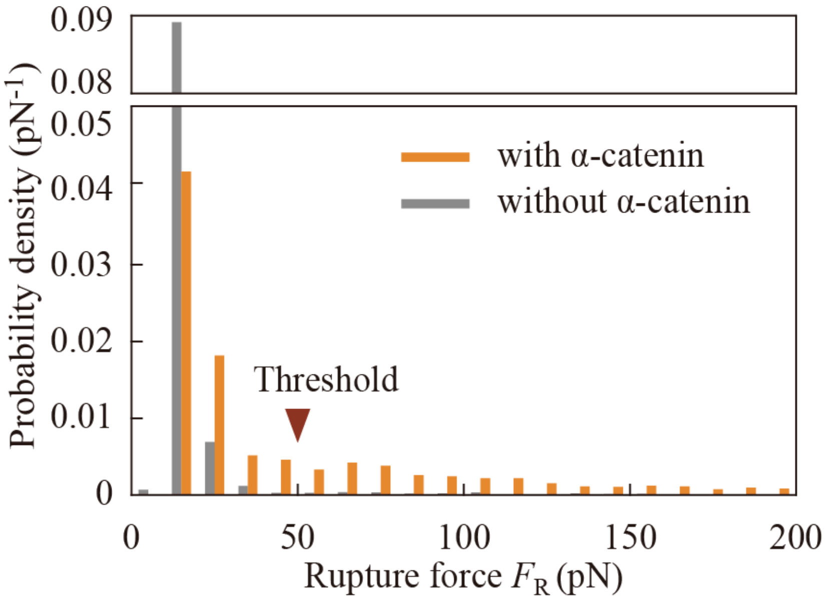

Sup. Fig. 1. Distributions of rupture force F_R for the experiments using α-catenin-modified coverslips (orange bars) and non-modified coverslips (gray bars; any other component were modified similarly.

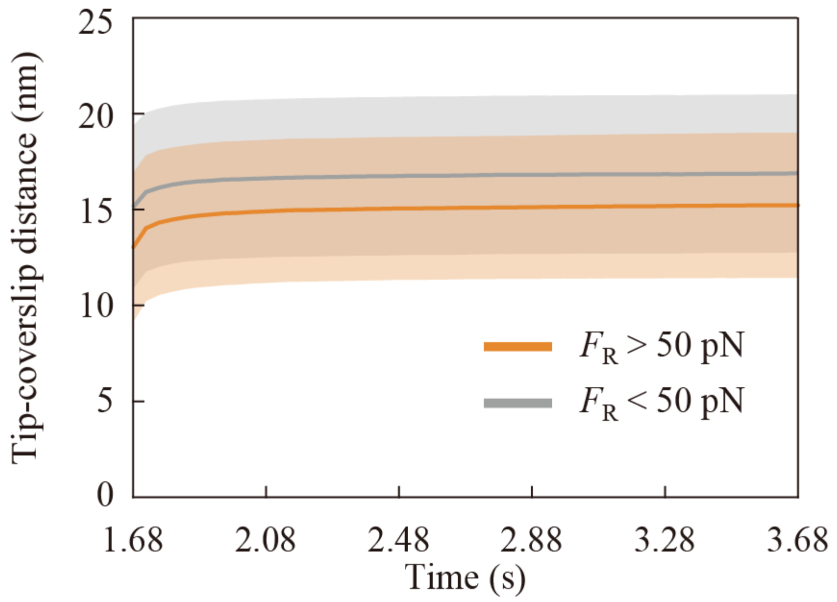

Sup. Fig. 2. Averaged tip-sample distances for the group with successful α-catenin extension (orange line) and without (gray line). The full widths at half maximum are shown as areas with light colors.

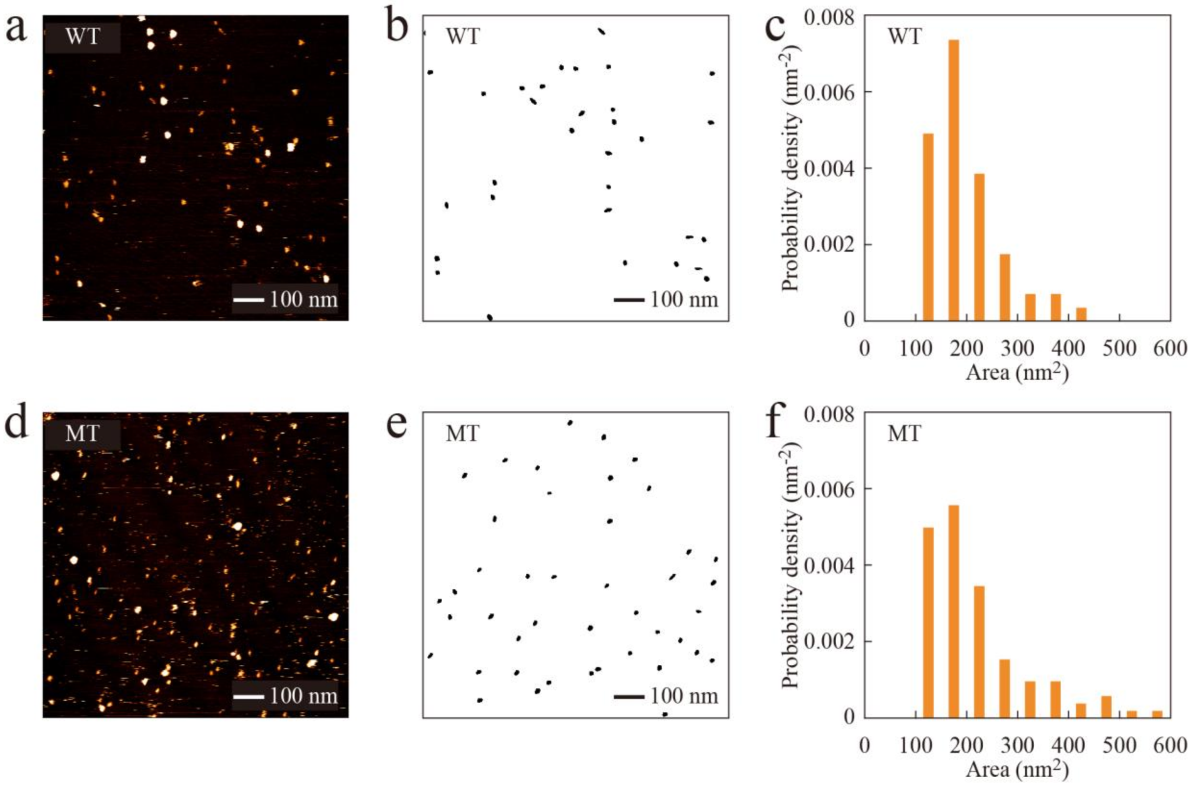

Sup. Fig. 3. AFM structural imaging of wild type (WT) and mutant (MT, M319G and R326E) α-catenin M_1-M_3 fragments. **(a, d)** AFM images of WT and MT α-catenin fragments in the working buffer containing DTT. The color contour is set from dark brown (0 nm) to white (3 nm) based on height. **(b, e)** Particle analysis. Raw images were binarized based on a threshold of 0.35 nm in height, and particles with more than 30 pixels, corresponding with ~114.4 nm^2, were analyzed. **(c, f)** Histograms of the area of particles. The mode value was in the range of 150 nm^2 to 200 nm^2 for both fragments. For reference, a spherical particle with a diameter of 4 nm is detected as a circle with area of 176.4 nm^2 in AFM imaging, when the curvature radius of the AFM tip is 7 nm.

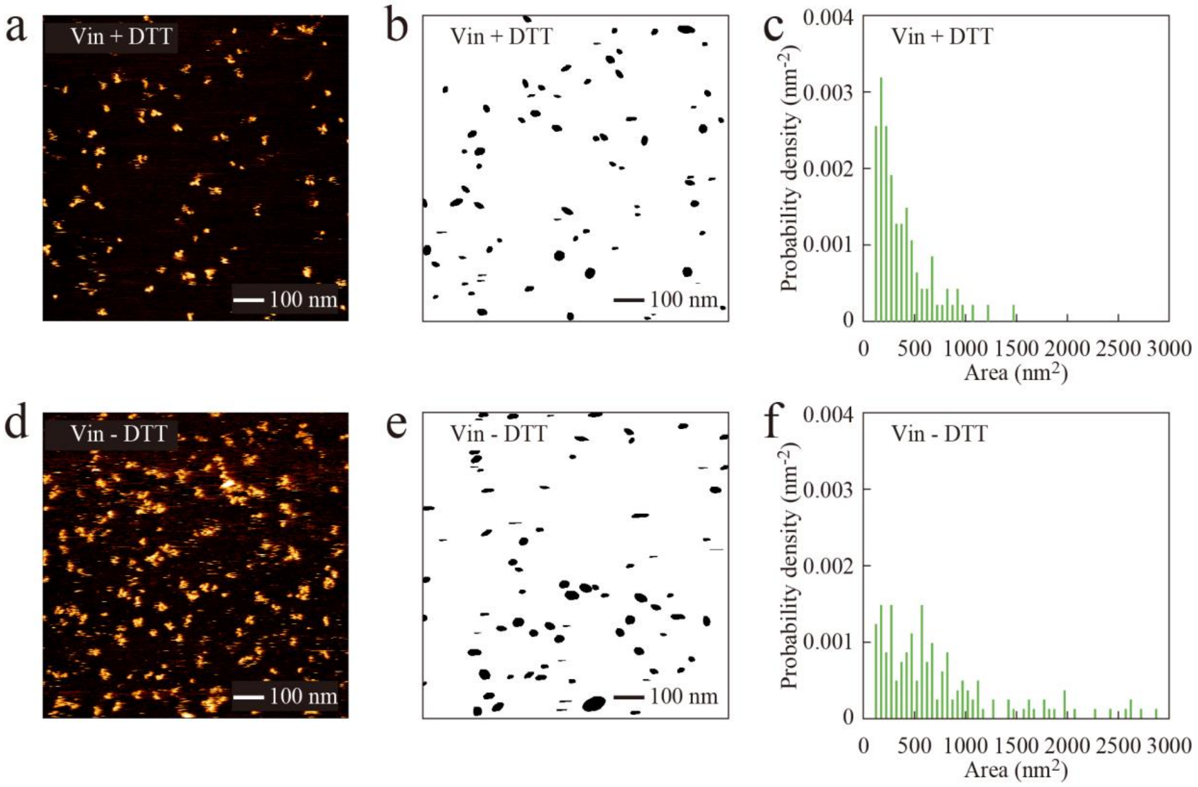

Sup. Fig. 4. AFM structural imaging of full-length vinculin. **(a, d)** AFM images of full-length vinculin in the working buffer containing DTT or not. The color contour is set from dark brown (0 nm) to white (3 nm) based on height. **(b, e)** Particle analysis. Detected particles are shown in black. **(c, f)** Histograms of area of particles. We verified that the addition of DTT increased the number of vinculin molecules that existed as the monomer in the working buffer.
